# Prediction of drug–drug interaction potential mediated by transporters between dasatinib and metformin, pravastatin, and rosuvastatin using physiologically based pharmacokinetic modeling

**DOI:** 10.1007/s00280-021-04394-z

**Published:** 2022-02-11

**Authors:** Ming Chang, Sai Bathena, Lisa J. Christopher, Hong Shen, Amit Roy

**Affiliations:** Bristol Myers Squibb, Princeton, NJ 08540 USA

**Keywords:** Dasatinib, PBPK model, Drug–drug interactions, Simcyp^®^

## Abstract

**Purpose:**

Recent in vitro studies demonstrated that dasatinib inhibits organic cation transporter 2 (OCT2), multidrug and toxin extrusion proteins (MATEs), and organic anion transporting polypeptide 1B1/1B3 (OATP1B1/1B3). We developed a physiologically based pharmacokinetic (PBPK) model to assess drug–drug interaction (DDI) potential between dasatinib and known substrates for these transporters in a virtual population.

**Methods:**

The dasatinib PBPK model was constructed using Simcyp^®^ Simulator by combining its physicochemical properties, in vitro data, in silico predictions, and pharmacokinetic (PK) results from clinical studies. Model validation against three independent clinical trials not used for model development included dasatinib DDI studies with ketoconazole, rifampin, and simvastatin. The validated model was used to simulate DDIs of dasatinib and known substrates for OCT2 and MATEs (metformin) and OATP1B1/1B3 (pravastatin and rosuvastatin).

**Results:**

Simulations of metformin PK in the presence and absence of dasatinib, using inhibitor constant (*K*_*i*_) values measured in vitro, produced estimated geometric mean ratios (GMRs) of the maximum observed concentration (C_max_) and area under the concentration–time curve (AUC) of 1.05 and 1.06, respectively. Sensitivity analysis showed metformin exposure increased < 30% in both AUC and C_max_ when dasatinib *K*_*i*_ was reduced by tenfold for OCT2 and MATEs simultaneously, and < 40% with a 20-fold *K*_*i*_ reduction. The estimated GMRs of C_max_ and AUC for pravastatin and rosuvastatin with co-administration of dasatinib were unity (1.00).

**Conclusions:**

This PBPK model accurately described the observed PK profiles of dasatinib. The validated PBPK model predicts low risk of clinically significant DDIs between dasatinib and metformin, pravastatin, or rosuvastatin.

**Supplementary Information:**

The online version contains supplementary material available at 10.1007/s00280-021-04394-z.

## Introduction

Dasatinib is a tyrosine kinase inhibitor (TKI) approved for the treatment of adult patients with Philadelphia-positive (Ph +) chronic myeloid leukemia (CML) or Ph + acute lymphoblastic leukemia (ALL) with resistance or intolerance to prior therapies including imatinib [1]. The recommended dosages of dasatinib are 100 mg and 140 mg administered orally once daily (QD) for patients with chronic phase CML and for patients with advanced phase CML and Ph + ALL, respectively [1]. Final results from the DASISION trial (NCT00481247) support the use of dasatinib 100 mg QD for the long-term treatment of CML in chronic phase [2].

Dasatinib pharmacokinetics (PK) are characterized by absorption following oral administration in patients with CML, and exposures are approximately dose proportional over a dose range of 15–240 mg [1]. The median time (minimum–maximum) to reach maximum observed concentration (T_max_) was 0.5 (0.25–1.5) h post-dose [3]. The overall mean elimination half-life of dasatinib is 3–5 h and is not affected by dosing regimen (QD or twice daily) or by disease status (CML in chronic or acute phase) [1]. Excretion of dasatinib is primarily via the fecal route (85%) with a small amount recovered in urine [3]. Approximately 80% of dasatinib metabolism is mediated by cytochrome P450 3A4 (CYP3A4) [4]. In vitro studies indicate that dasatinib is a time-dependent inhibitor of CYP3A4, but has little potential to induce CYP enzymes [5, 6]. Additionally, in vitro studies have also demonstrated that dasatinib is likely a weak substrate and not an inhibitor of P-glycoprotein (P-gp) [7].

The important role of transporters in drug safety and efficacy has become increasingly clear over the past few decades, and has had a substantial impact on drug development and medicine [8–10]. Recently, transporter-mediated drug–drug interaction (DDI) potential for dasatinib as an inhibitor was evaluated in cells overexpressing membrane transporters (Table 1 and Supplementary Table 1). Dasatinib was shown to inhibit organic cation transporter 2 (OCT2), multidrug and toxin extrusion proteins (MATE1/2K), and organic anion transporting polypeptide 1B1/1B3 (OATP1B1/1B3) with half maximal inhibitory concentration (IC_50_) values of 0.034, 0.22, 0.86, 9.2, and 4.4 μM, respectively. Based on these in vitro findings, we investigated whether dasatinib has potential inhibitory effects on OCT2, MATEs (MATE1/2K), and OATP1B1/1B3 transporters in vivo.

**Table 1:** Input parameters of dasatinib for PBPK model in Simcyp^®^ Simulator (v18.1)

Parameters	Value	Data source/comment
**Physicochemical parameters**		
Molecular weight	488.01	Sprycel Package Insert [1]
LogP	3.20	Predicted in Simcyp^®^ from LogD (4.27–3.23, pH 2–9)
pKa	3.1, 6.8	DCN950068845 (Supplementary Table 1)
Compound type	Diprotic base	
Blood/plasma ratio	1.8	Kamath et al. 2008 [7]
fu in plasma	0.04	NDA clinical pharmacology summary
**Absorption (ADAM model)**		
*f*_*a*_	0.9996	Predicted in Simcyp^®^ from MechP_eff_ model
ka (h^−1^)	4.226	Predicted in Simcyp^®^ from MechP_eff_ model
fu_Gut_	0.04	fu in plasma
Effective human Pc (10^−4^ cm/s)	9.667	Predicted in Simcyp^®^ from MechP_eff_ model
Particle size (µm)	38 (average)	DCN950068845 (Supplementary Table 1); 50 mg tablet
Intrinsic solubility (mg/mL)	0.03	DCN950068845 (Supplementary Table 1); Bio-relevant solubility
**Distribution (full PBPK model)**		
Kp scaler	0.7	Optimized to fit the observed PK profiles
Vss (L/kg)	5.2	Predicted using method 2 full PBPK model in Simcyp^®^
**Elimination**		
CLpo (L/h)	338	Averaged from 4 studies (CA180009, CA180016, CA180032, and CA180249; BMS, Supplementary Table 1)
CYP3A4 CLint (µL/min/pmol CYP)	11.635	Predicted in Simcyp^®^ retrograde when CYP3A4 accounts for 82.5% with *f*_*a*_ of 0.8 (DCN930011322; Supplementary Table 1)
Renal clearance (L/h)	0.4	Human ADME CA180019 [3]
Additional HLM CLint (µL/min/mg protein)	319.32	Predicted in Simcyp^®^ retrograde
**Transporter**^a^	n.a	
**Interaction**^b,c^		
CYP3A4 *K*_*i*_ (µM)	9.0 (midazolam) 5.0 (testosterone)^c,d^	DCN930011322 (Supplementary Table 1)
CYP3A4 *K*_app_ (µM)	1.9	DCN930011322 (Supplementary Table 1)
CYP3A4 *K*_inact_ (min^*−1*^)	0.022 (midazolam)	DCN930011322 (Supplementary Table 1)
CYP2C8 *K*_*i*_ (µM)	3.6	DCN930011322 (Supplementary Table 1)
OCT2 *K*_*i*_ (µM)	0.034	DCN930147497 (Supplementary Table 1)
MATE1 *K*_*i*_ (µM)	0.22	
MATE2K *K*_*i*_ (µM)	0.86	DCN930147085 (Supplementary Table 1)
OATP1B1 *K*_*i*_ (µM)	9.2 (2.33)^d^	DCN930147497 (Pahwa et al. 2017 [16]; Supplementary Table 1)
OATP1B3 *K*_*i*_ (µM)	4.4 (2.75)^d^	

*ADAM* advanced dissolution, absorption, and metabolism, *ADME* absorption, distribution, metabolism, and excretion, *BCRP* breast cancer resistance protein, *BMS* Bristol Myers Squibb*, CLint* intrinsic clearance, *CLpo* oral clearance, *CYP* cytochrome P450, *f*_*a*_ fraction absorbed, *fu* fraction unbound, *fu*_*Gut*_ fraction unbound in the gut, *HLM* human liver microsome, *Kp* partition coefficient, *IC*_*50*_ half maximal inhibitory concentration, *ka* first-order absorption rate constant, *K*_*app*_ concentration of mechanism-based inhibition at 50% *K*_inact_, *K*_*i*_ inhibitor constant, *K*_*inact*_ maximal inactivation rate, *K*_*m*_ amount of substrate needed to reach half of the maximum velocity of the reaction, *MATE* multidrug and toxin extrusion protein, *MRP* multidrug resistance-associated protein, *n.a.* not available, *NDA* new drug application, *OAT* organic anion transporter, *OATP* organic anion transporting polypeptide, *OCT* organic cation transporter, *PBPK* physiologically based pharmacokinetic, *Pc* permeability, *P-gp* P-glycoprotein, *PK* pharmacokinetic, *pKa* acid dissociation constant, *S* substrate concentration, *Vss* volume of distribution at steady state

^a^The in vitro information of dasatinib as transporter substrate was not available

^b^Except for CYP3A4 *K*_app_ and CYP3A4 *K*_inact_, *K*_i_ was calculated from IC_50_ using the equation for competitive inhibition (*K*_i_ = IC_50_/[(S/*K*_m_) + 1]

^c^Dasatinib was not expected to inhibit other CYP enzymes or transporters; the IC_50_ values for other CYPs (CYP1A2, CYP2A6, CYP2B6, CYPC9, CYP2C19, CYP2D6, and CYP2E1) were ≥ 35 µM and values for other transporters (P-gp, BCRP, MRP2, MRP3, MRP4, OAT1, and OAT3) were ≥ 19.5 µM

^d^When more than one *K*_i_ value was reported/listed, the model used the most potent value for a conservative approach, i.e., 5.0 (testosterone) versus 9.0 µM (midazolam) for CYP3A4; 2.33 and 2.75 versus 9.2 and 4.4 µM for OATP1B1 and OATP1B3, respectively

Previous studies have shown that physiologically based pharmacokinetic (PBPK) modeling enables assessment of the DDI potential of drugs in the absence of clinical data [11–13]. Therefore, we developed a PBPK model in a virtual population to assess transporter-mediated interaction potential between dasatinib and known substrates for OCT2 and MATEs (metformin) and OATP1B1/1B3 (pravastatin and rosuvastatin). A PBPK model for dasatinib was developed within Simcyp^®^ Simulator based on physicochemical properties and in vitro data, and verified using additional clinical PK and DDI observations for dasatinib. Sensitivity analyses provided further assessment of DDI potential by examining key parameters that may impact model-predicted DDI effects.

## Materials and methods

### Overall modeling procedure

Clinical data used in model development are summarized in Supplementary Table 2. The clinical studies were conducted in accordance with the Declaration of Helsinki and the International Council for Harmonisation Guideline for Good Clinical Practice and approved by ethics committees.

**Fig. 1:**
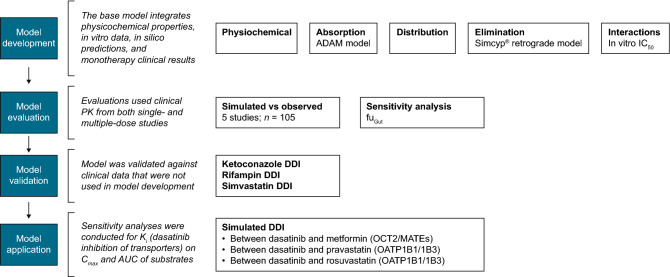
PBPK modeling workflow. *ADAM* advanced dissolution, absorption, and metabolism, *AUC* area under the time–concentration curve, *CLpo* oral clearance, *C*_*max*_ maximum observed concentration, *CYP3A4* cytochrome P450 3A4, *DDI* drug–drug interaction, *fu*_*Gut*_ fraction unbound in the gut, *IC*_*50*_ half maximal inhibitory concentration, *K*_*i*_ inhibitor constant, *MATE* multidrug and toxin extrusion protein, *OATP* organic anion transporting polypeptide, *OCT* organic cation transporter, *PBPK* physiologically based pharmacokinetic. The Simcyp^®^ Simulator (version 18.1, Certara, Princeton, USA) was used for model development, validation, and applications. Intrinsic clearances of CYP3A4 and other enzymes were predicted based on total body clearance (CLpo) observed following oral administration and percentage of enzymes responsible for dasatinib metabolism determined in vitro. Sensitivity analyses of fu_Gut_ on the C_max_ and AUC of dasatinib were conducted to optimize the value for modeling input

The modeling strategy for the PBPK model of dasatinib, including model development, evaluation, validation, and application, are shown in Fig. 1.

#### Model development

The development of the dasatinib PBPK model employed a stepwise “middle-out” approach to estimate the human PBPK parameters for dasatinib using Simcyp^®^ Simulator (version 18.1, Certara, Princeton, USA). The base model was constructed by integrating the physicochemical properties, in vitro experimental data, in silico predictions, and clinical results of monotherapy studies. A mechanistic advanced dissolution, absorption, and metabolism (ADAM) model was used to describe absorption and a full PBPK distribution model described the distribution. Elimination intrinsic clearances of CYP3A4 and other enzymes were predicted by the retrograde method (a “top-down” approach to derive clearance from clinical PK) within Simcyp^®^ based on both the oral clearance (CLpo) observed following oral administration in human and the fraction (fm) of metabolites formed by CYP3A4 enzymes responsible for dasatinib metabolism determined in vitro.

#### Model evaluation

The base model for dasatinib was evaluated with clinical PK data from four single-dose (100 mg QD) and one multiple-dose study, listed in Supplementary Table 2. The simulated versus observed mean plasma concentration–time profiles and key PK parameters (maximum observed concentration [C_max_] and area under the concentration–time curve [AUC]) were compared using the four single-dose studies. The model input parameters that were not directly measured, such as *f*_*a*_ (fraction absorbed, used in the Simcyp^®^ retrograde calculator) and partition coefficient (Kp) scalars (used to adjust the tissue-to-plasma Kp values), were adjusted to fit the observations. In addition, overlay plots of simulated and observed plasma concentration–time profiles following single- and multiple-dose administration were visually evaluated. Given the uncertainty of the unbound fraction in the gut (fu_Gut_), which was not directly measured, sensitivity analyses of fu_Gut_ on the C_max_ and AUC of dasatinib were also conducted to optimize the value for modeling input.

#### Model validation

The dasatinib base model was validated against clinical data from three independent DDI studies that were not used in model development. These definitive clinical DDI trials evaluated interaction potential between dasatinib and ketoconazole (a strong CYP3A inhibitor), rifampin (a strong CYP3A inducer), and simvastatin (a CYP3A4 substrate) (Supplementary Table 1). The simulated versus observed results were compared by overlaid plasma profiles, geometric mean ratios (GMRs) of C_max_ and AUC, and plots of GMRs from virtual studies versus corresponding clinical trials.

#### Model application

The validated PBPK dasatinib model was used to assess the DDI potential of dasatinib as a perpetrator drug in the clinically untested scenarios of DDI mediated by (1) OCT2 and MATE transporters and (2) OATP1B1/1B3 transporters. With metformin as an example substrate for OCT2 and MATEs, the DDI simulation was performed using the electrochemical gradient-driven (EGD) model in healthy subjects. Two example substrates, pravastatin and rosuvastatin, were used to predict OATP1B1/1B3–based DDI. As a metric of potential DDI, the GMRs of C_max_ and AUC for each of the example substrates in the presence or absence of dasatinib were evaluated.

Parameter sensitivity analyses were performed for the in vitro inhibitory constant (*K*_*i*_) values of dasatinib against the transporters. The following scenarios for modeling inputs were tested: scenario 1 used the measured in vitro *K*_*i*_ values of dasatinib for the transporters, scenario 2 used more potent inhibition with *K*_*i*_ values reduced tenfold, and scenario 3 (only for OCT2 and MATEs) used *K*_*i*_ values reduced 20-fold.

#### Modeling software and simulation design

The dasatinib model was constructed based on physicochemical properties, measured in vitro data, and dasatinib clinical results. The default compound and population library files within Simcyp^®^ were used without further modification, except for the simvastatin compound file, for which a 40% reduction in human liver microsome (HLM) elimination was incorporated. The adjustment was to fit the CLpo value derived using in-house data from a dasatinib–simvastatin clinical DDI study (Supplementary Table 1). Specifically, CYP3A4 intrinsic clearance (CLint) was reduced from 2284 to 1370 μL/min/pmol CYP, and predicted enzyme CLint was reduced from 254 to 152 μL/min/pmol of enzyme. The input parameters used in Simcyp^®^ simulations are shown in Table 1.

Key assumptions for the PBPK modeling were: (1) there was a negligible effect of P-gp efflux to reduce dasatinib absorption in the gastrointestinal tract, (2) biliary excretion of unchanged dasatinib was negligible, and (3) *K*_*i*_ values, required as input parameters for inhibition of CYP enzymes and membrane transporters, were simplified as *K*_*i*_ = IC_50_/2 for enzyme-based reactions and *K*_*i*_ = IC_50_ for transporter-based inhibition. Using measured IC_50_ values, *K*_*i*_ was calculated according to the equation for competitive inhibition, *K*_*i*_ = IC_50_/([S/*K*_m_] + 1), where S is the initial substrate concentration in the experiment, and *K*_m_ is the amount of substrate needed to reach half of the maximum velocity of the reaction (*V*_max_). Assays characterizing CYP enzymes generally used initial substrate concentrations approximating *K*_*m*_, resulting in *K*_*i*_ = IC_50_/2. Assays characterizing transporters generally used substrate concentrations much lower than *K*_*m*_ (S << *K*_*m*_), so *K*_*i*_ = IC_50_. The simulation design information for each modeling stage is summarized in Supplementary Table 3.

## Results

### Model evaluation

#### Simulation results for single-dose PK of dasatinib

The simulated and observed mean plasma profiles of dasatinib following a 100-mg single-dose administration are shown in Supplementary Fig. 1. The model was calibrated to ensure the simulated concentration–time profiles were in good agreement with the observed profiles measured in healthy adults (Supplementary Table 4). A sensitivity analysis was performed to evaluate the effect of fu_Gut_ on the estimated C_max_ and AUC of dasatinib. Tested values for fu_Gut_ ranged from 0 to 1, including three values typically used: 0.00173 (the value predicted by Simcyp^®^ based on the physical–chemical properties of dasatinib, 0.04 (plasma free fraction [fu_plasma_]), and 1.0 (this value predicted the worst-case scenario, if no information is available). Based on the sensitivity analysis, there was best agreement between the predicted PK and observed dasatinib PK when the fu_Gut_ was set at 0.04, equivalent with fu_plasma_.

#### Simulation results for multiple-dose PK of dasatinib

Simulated and observed mean plasma profiles following dasatinib 75 mg QD multiple-dose administration (5 days on and 2 days off) are shown in Supplementary Fig. 2. The simulated PK profiles matched the clinical data obtained from three patients, suggesting that the model reasonably captured the monotherapy data following the repeat QD dosing.

### Model validation

The PBPK model was validated by three independent DDI studies that were not used for model building (Supplementary Table 1). The simulated results were in good agreement when compared with observations from three definitive clinical DDI trials: ketoconazole (CA180021), rifampin (CA180032), and simvastatin (CA180022) (Supplementary Table 1, Supplementary Table 5, and Supplementary Fig. 3).

### Model application: prediction of clinically untested DDI for dasatinib as a perpetrator

#### Prediction of effect of dasatinib on metformin PK

In vitro experiments showed that dasatinib inhibited renal transporters OCT2, MATE1, and MATE2K with *K*_*i*_ values of 0.034, 0.22, and 0.86 μM, respectively. Using metformin as an example substrate for the renal transporters, the model predicted potential transporter-mediated DDI with dasatinib. As Simcyp^®^ allowed only a single value input for the MATE transporters, the lower (more potent) *K*_*i*_ value of 0.22 μM determined for MATE1 was selected as the input parameter.

**Fig. 2:**
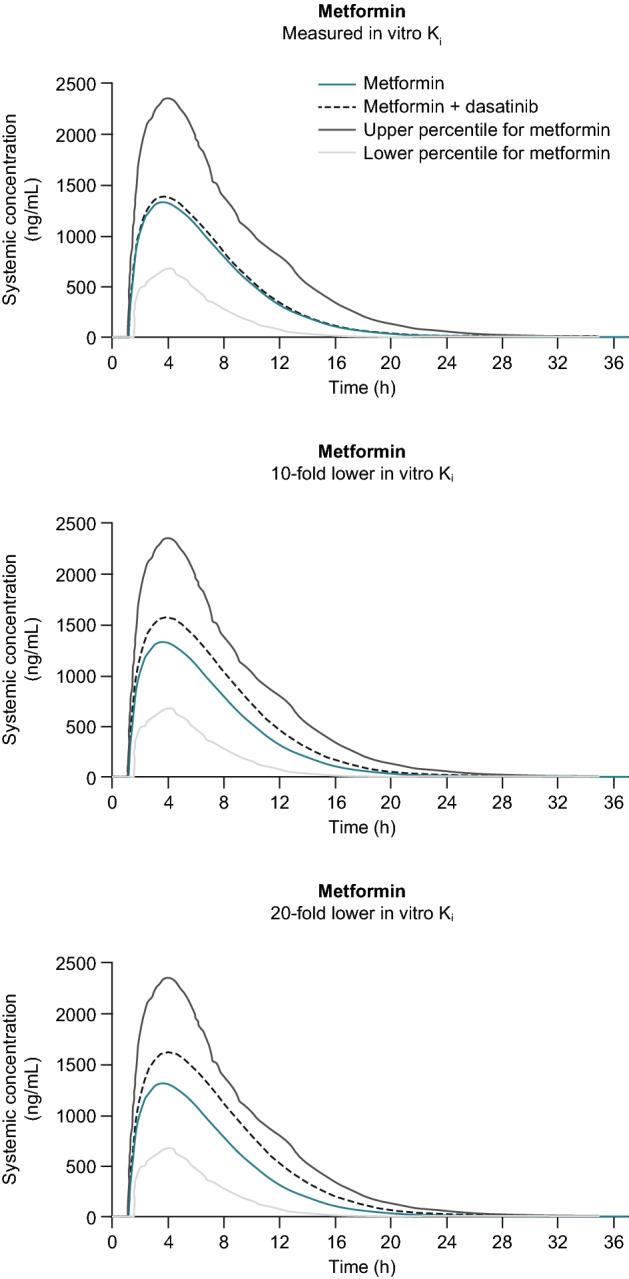
Predicted mean plasma concentration–time profiles of metformin. *K*_*i*_ inhibitor constant

Using measured in vitro *K*_*i*_ values (scenario 1), the estimated GMRs of C_max_ and AUC were 1.05 and 1.06, respectively, indicating a 5–6% increase in metformin exposure if metformin and dasatinib are used concomitantly (Table 2, Fig. 2). Sensitivity analyses showed that metformin exposure increased by 20% and 28% in C_max_ and AUC, respectively, if dasatinib in vitro *K*_i_ was reduced by tenfold for OCT2 and MATEs simultaneously (scenario 2), and increased by 25% and 39%, respectively, with a 20-fold *K*_*i*_ reduction (scenario 3). The predicted values suggest that metformin exposures increase by < 40% if the two drugs are used in combination.

**Table 2:** GMR of C_max_ and AUC for metformin, pravastatin, and rosuvastatin when co-administered with dasatinib versus administered alone

Treatment	Scenario	GMR of C_max_ (90% CI)	GMR of AUC (90% CI)
Metformin	Measured in vitro *K*_*i*_		
	OCT2 (0.034 µM)/MATEs (0.22 µM)	1.05 (1.04–1.05)	1.06 (1.05–1.06)
	Tenfold lower *K*_*i*_		
	OCT2 (0.0034 µM)/MATEs (0.022 µM)	1.20 (1.18–1.22)	1.28 (1.26–1.30)
	20-fold lower *K*_*i*_		
	OCT2 (0.0017 µM)/MATEs (0.011 µM)	1.25 (1.23–1.28)	1.39 (1.36–1.42)
Pravastatin	Reported in vitro *K*_*i*_		
	OATP1B1 (2.4 µM)/OATP1B3 (2.88 µM)	1.00 (1.00–1.00)	1.00 (1.00–1.00)
	Tenfold lower *K*_*i*_		
	OATP1B1 (0.24 µM)/OATP1B3 (0.288 µM)	1.00 (1.00–1.00)	1.00 (1.00–1.00)
Rosuvastatin	Reported in vitro *K*_*i*_		
	OATP1B1 (2.4 µM)/OATP1B3 (2.88 µM)	1.00 (1.00–1.00)	1.00 (1.00–1.00)
	Tenfold lower *K*_*i*_		
	OATP1B1 (0.24 µM)/OATP1B3 (0.288 µM)	1.00 (1.00–1.00)	1.00 (1.00–1.00)

*AUC* area under the concentration–time curve, *CI* confidence interval, *C*_*max*_ maximum observed concentration, *K*_*i*_ inhibitor constant, *GMR* geometric mean ratio, *MATE* multidrug and toxin extrusion protein, *OATP* organic anion transporting polypeptide, *OCT* organic cation transporter

#### Prediction of effect of dasatinib on pravastatin and rosuvastatin PK

In vitro experiments showed that dasatinib inhibited liver transporters OATP1B1 and OATP1B3 with IC_50_ values of 9.18 μM and 4.36 μM, respectively (literature-reported values are 2.33 μM and 2.75 μM, respectively [14]). The most potent values were applied to the model as a conservative approach, i.e., 2.33 μM and 2.75 μM for OATP1B1 and OATP1B3, respectively. The estimated GMRs of C_max_ and AUC for pravastatin and rosuvastatin in the presence and absence of dasatinib were unity (1.00; Table 2, Supplementary Fig. 4). Additionally, sensitivity analysis showed that for both example substrates pravastatin and rosuvastatin, tested under the two *K*_*i*_ scenarios, the estimated GMRs of C_max_ and AUC were unity (1.00). These data demonstrate that co-administration of dasatinib caused little impact on the PK of pravastatin or rosuvastatin through inhibition of OATP1B1/1B3 transporters.

## Discussion

### Model development

Dasatinib is classified as a Biopharmaceutics Classification System class II compound and the aqueous solubility showed a significant decrease as pH increased [15, 16]. The ADAM model, used to simulate dasatinib oral absorption, incorporated pH-dependent solubility and formulation, and was able to predict human intestinal effective permeability (P_eff_) through the MechP_eff_ model. In vitro studies suggest that dasatinib might be a substrate of P-gp with an efflux ratio (B-A/A-B) in Caco-2 cell monolayers slightly greater than 2 (22.2/10.2 10^−6^ cm/s) [7]. However, in clinical studies, dasatinib exhibited linear PK over the dose range of 15–240 mg/day in both patient and healthy populations, suggesting that the impact of P-gp on dasatinib PK was likely to be minimal. Additionally, dasatinib exhibits high permeability in Caco-2 assays, allowing the model to reflect accurately the clinical data with respect to the rate of absorption. Therefore, P-gp was not included in the current dasatinib model. The simulated PK profiles matched observed data from multiple independent studies, demonstrating that the assumption was reasonable.

The elimination model built in Simcyp^®^ used CLpo, as human PK data after intravenous (IV) dosing were not available. The 100-mg dose was selected for modeling experiments because most of the PK data were generated from phase I studies using this dose. The model input value of CLpo (338 L/h) was a calculated mean of CLpo values from 102 healthy adults from four independent trials who received a single oral dose of 100-mg dasatinib under fasted conditions. The intrinsic metabolic clearance values for CYP enzymes were estimated by the retrograde method in Simcyp^®^, and these input values were slightly optimized to reflect accurately the clinical PK data with regard to C_max_ and AUC that were derived from the same population of 102 healthy adults.

Based on human absorption, distribution, metabolism, and excretion (ADME) data, which indicated < 20% recovery of parent drug in the urine and feces combined [3], the model assumed that metabolic clearance was the dominant pathway for dasatinib disposition, with a negligible contribution from biliary excretion as unchanged parent drug. Generally, biliary excretion involves active secretion of drug molecules or their metabolites from hepatocytes into the bile, and then the gut, where the drugs are excreted. Without the specific knowledge of biliary excretion, efflux transporters, and clearance following IV dosing, the biliary clearance of dasatinib was considered a non-identifiable parameter in model development. The simulated outcome fits well with observed data providing support for the assumption of minimal biliary excretion of dasatinib.

### Model evaluation and validation

As an input parameter in Simcyp^®^ Simulator, fu_Gut_ was used in the equation as an element together with flow in the gut (Q_gut_) and intrinsic metabolic clearance in the gut (CLu_int-gut_) to calculate the gut extraction/metabolism (Fg) of a drug. Although the value of fu_Gut_ is not routinely measured, it can be evaluated in the Simcyp^®^ model with some basic considerations, such as physical–chemical properties of a drug, and sensitivity testing. When testing fu_Gut_, there are five scenarios that are generally considered [17]: (1) equilibrating with free concentration in plasma; (2) equilibrating with free concentration in the blood; (3) involving an active uptake component for uptake into the enterocytes; (4) utilizing a value predicted by Simcyp^®^ based on the physical–chemical properties of the drug; and (5) using a value of 1 to predict the worst-case scenario, if no information is available.

Among the five scenarios, active uptake into enterocytes for dasatinib was not found and unbound fraction in blood (fu_blood_) was not measured. Since sensitivity analysis is an important step to assess modeling uncertainty, it was performed to look at the impact of fu_Gut_ on the C_max_ and AUC of dasatinib. The sensitivity analyses demonstrated that the value of 1 simulated 50% of observed dasatinib C_max_ and AUC, indicating that the input value of 1 for the worst-case-scenario under-predicted the dasatinib PK. Between the values predicted by the model (0.00173) and fu_plasma_ (0.04), the value of 0.04 gave the best agreement with the observed data in both PK parameters, indicating that fu in enterocytes being in equilibrium with fu_plasma_ was a reasonable assumption. The prediction was also consistent with the profile of low-solubility and high permeability for dasatinib. Therefore, results from the sensitivity analysis were used to set up the input value for fu_Gut_. The model performance was validated by three independent DDI studies. For all three studies, the simulated results were in good agreement with observed clinical data, verifying the PK pathways assigned to the PBPK model for dasatinib. The GMRs of the simulated versus observed values for both AUC and C_max_ (0.76–1.07) were within a twofold range, indicating satisfactory predictive performance of the dasatinib model. The Simcyp^®^ library compound files were used without modification except for simvastatin. The rationale for modifying the simvastatin library file was that it poorly described the simvastatin PK profile observed from an in-house clinical study of simvastatin dosed alone in healthy adults (Supplementary Table 1). Based on the CLpo value reported in the clinical study report, the clearance in the library file decreased by 40%. The simulated results were in good agreement with observed data from three definitive clinical DDI trials, demonstrating a reasonable predictive capability of the PBPK model for dasatinib.

### Model application

**Fig. 3:**
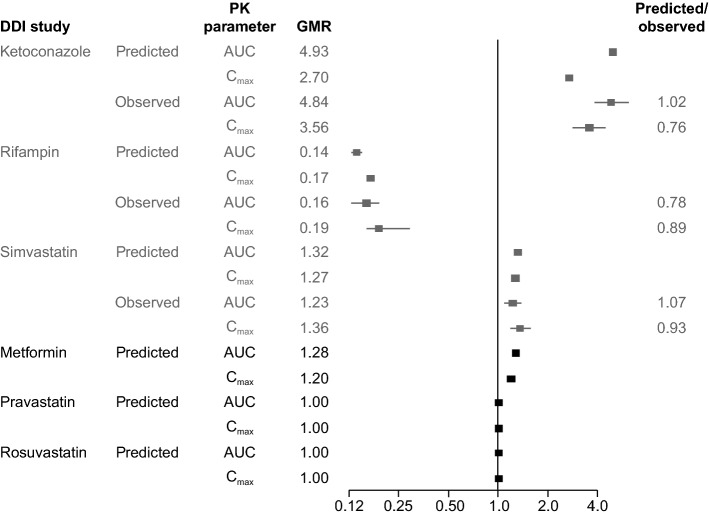
Forest plot summarizing predicted and observed (if available) GMRs of AUC and C_max_ in DDI studies. *AUC* area under the time–concentration curve, *C*_*max*_ maximum observed concentration, *DDI* drug–drug interaction, *GMR* geometric mean ratio, *PK* pharmacokinetic

The validated dasatinib model was used to predict DDIs mediated by transporters using metformin, pravastatin, and rosuvastatin as example substrates and dasatinib as a perpetrator drug (Fig. 3). Transporter-mediated DDI potential for dasatinib as an inhibitor was recently evaluated in cells overexpressing membrane transporters (Supplementary Table 1). The experiment included a full panel of transporters (P-gp, breast cancer resistance protein [BCRP], OATP1B1, OATP1B3, multidrug resistance-associated protein 2 [MRP2], organic anion transporter 1 and 3 [OAT1 and OAT3], OCT1, OCT2, MATE1, and MATE2K) that have been reported to have a clinically relevant impact on PK or pharmacodynamics, including those recommended by the US Food and Drug Administration (FDA) and European Medicines Agency DDI guidance. Among the 15 transporters tested, dasatinib did not inhibit P-gp, BCRP, MRP2, OAT1, and OAT3. However, potent inhibition was shown for OCT2, MATE1, MATE2K, OATP1B1, and OATP1B3 (Table 1 and Supplementary Table 1). These in vitro findings highlight the important question of whether dasatinib has an inhibitory effect in vivo on OCT2, MATEs (MATE1/MATE2K), and OATP1B1/1B3 transporters. Metformin is an example of an OCT2 substrate recommended by the FDA for use in clinical investigations of OCT2-mediated transporter DDIs; both pravastatin and rosuvastatin are FDA recommended as example substrates of OATP1B1 and OATP1B3.

#### DDI potential mediated by OCT2 and MATEs

Transport through OCT is reported to be dependent on the electrochemical gradient across the cell membrane, while MATE is a proton antiporter [18]. The EGD model developed by Simcyp^®^ reproduced DDI caused by cimetidine as an inhibitor for both OCT2 and MATE1 [19]. The EGD model recovered the observed AUC ratio of metformin in the presence or absence of cimetidine using *K*_*i*_ reduced 8- to 18-fold, compared with a 1000-fold reduction in *K*_*i*_ used in the Simcyp^®^ conventional model [20]. Therefore, in this study, the metformin EGD model was employed to predict the effect of dasatinib as an inhibitor of OCT2 and MATE transporters on metformin exposure.

However, there were marked differences between cimetidine and dasatinib regarding inhibitory profiles both in vitro and in vivo. Based on in vitro *K*_*i*_ values, MATE1 (1.1 µM) was the dominant contributor in cimetidine inhibition compared with OCT2 (124 µM) [21, 22], while dasatinib was more potent against OCT2 (0.034 µM) with 6.6-fold and 25-fold potency over MATE1 (0.225 µM) and MATE2K (0.86 µM), respectively.

In humans, following a clinically approved therapeutical dose of 100 mg dasatinib, the C_max_ of dasatinib in plasma averaged 80 ng/mL (164 nM). The maximal unbound fraction of dasatinib in circulation (C_max,u_) was 6.6 nM, which was much lower than the IC_50_ values for OCT2, MATE1, and MATE2K. In contrast, the C_max,u_ of cimetidine (approximately 3 µM) was comparable to or greater than the IC_50_ values of MATE1 (1.1 µM) and MATE2K (7.3 µM) [22, 23].

According to Ito et al., the underlying DDI mechanism of cimetidine is likely MATEs rather than OCT2 [21]. Reports from Pelis et al. also pointed out that inhibition of MATE transporters played a major role in the accumulation of high drug concentration in renal cells [18]. Together, the integrated in vitro and in vivo information may explain the difference in DDI between metformin and dasatinib versus cimetidine.

Additionally, kidney tissue concentration of dasatinib was evaluated based on the model predictions as observed data were not available. Assuming the free fraction of dasatinib in kidney tissue was similar to that in plasma, the PBPK model predicted the maximal C_max_ in kidney tissue to be 52 nM, which was comparable to IC_50_ for OCT2, but maintained a short time of < 1.5 h. This maximum concentration in kidney tissue was much lower than the IC_50_ values for MATE transporters. Therefore, strong DDI between metformin and dasatinib was unlikely to be expected. *K*_*i*_ or IC_50_ values, estimated from transcellular transport experiments, are considered uncertainty factors, mainly due to inter-laboratory variations including assay differences in cell lines, example substrates and concentrations used, and IC_50_ calculation methods [24]. These uncertainties impact the accurate prediction of transporter-mediated DDI risks. To attain confidence in model predictions, we conducted sensitivity analyses of IC_50_ (*K*_*i*_) over a broad range, using three *K*_i_ scenarios for dasatinib inhibition to report changes in metformin GMRs of AUC and C_max_ with or without dasatinib. Scenario 1 used the measured in vitro *K*_i_, providing a baseline value, and the 5–6% changes in GMRs of C_max_ and AUC indicated little impact of dasatinib on metformin PK after co-administration of the two drugs. In scenario 2, metformin C_max_ and AUC increased by 20% and 28%, respectively, when the two drugs were dosed concomitantly. The tenfold reduction in *K*_*i*_ of scenario 2 likely accounts for the typical variations in experimental assays for the two transporters reported elsewhere [25]. Evaluation of 20-fold lower *K*_*i*_ (scenario 3) follows from the study of Burt et al., in which an 8- to 18-fold decrease in cimetidine *K*_*i*_ for both OCT2 and MATEs reproduced the effect of cimetidine on metformin exposure observed in clinical study results [20]. Our results show that both C_max_ and AUC ratios increased as OCT2 and MATE inhibition potency increased (Fig. 2). With a 20-fold reduction in *K*_i_ simultaneously for both OCT2 and MATEs, the increases in metformin C_max_ and AUC were 25% and 39%, respectively. The PK changes of < 40% were not expected to be clinically meaningful, because metformin is a well-tolerated drug based on the DDI study by Zack et al. [26].

Among the drugs that are substrates of OCT2 and MATE transporters, concomitant medications commonly prescribed to patients receiving TKIs (such as imatinib, dasatinib, and nilotinib) include ranitidine and lamivudine, as well as metformin [27]. Similar to metformin [26], ranitidine [28] and lamivudine [29] have good safety profiles, and as such, concurrent administration of dasatinib is unlikely to have an effect on safety.

#### DDI potential mediated by OATP1B1 and OATP1B3

Pravastatin and rosuvastatin, both recommended by the FDA as example substrates of OATP1B1 and OATP1B3, were employed in this study in two separate simulations. The Simcyp^®^ library compound files of pravastatin and rosuvastatin were used without modification. The *K*_*i*_ (IC_50_) values generated in-house for dasatinib inhibition of hepatic transporters OATP1B1 (9.18 μM) and OATP1B3 (4.36 μM) differed slightly from reported values of 2.33 μM and 2.75 μM [14], respectively, and we used the more potent values for prediction. As with the metformin simulation, sensitivity analysis on *K*_*i*_ was performed, with scenario 1 using the measured in vitro *K*_*i*_ and scenario 2 using a tenfold lower *K*_i_ for both OATP1B1 and OATP1B3, simultaneously. The simulation results showed that GMRs of C_max_ and AUC for both pravastatin and rosuvastatin in the presence and absence of dasatinib were unity. Sensitivity analyses also confirmed little change in C_max_ and AUC ratios as OATP inhibition potency increased. The minimal impact was likely due, in part, to low dasatinib exposure on the targeted liver tissue (C_max_ of 0.67 μM in the portal vein). The results demonstrated a low risk of DDI mediated by OATP1B1/1B3 transporters via dasatinib inhibition.

## Conclusion

A PBPK model for dasatinib was developed and validated with several independent clinical DDI studies. The model was able to describe the observed PK profiles of dasatinib and simulated drug interactions appropriately. The validated PBPK model predicted a low potential for clinically significant DDIs between dasatinib and metformin, pravastatin, or rosuvastatin through inhibition of OCT2, MATEs, and OATP1B1/1B3 transporters. The predicted results suggest < 40% increase in metformin exposure and little change in pravastatin and rosuvastatin exposure if given together with dasatinib. The clinical significance of these interactions is deemed minimal. Therefore, no major changes in PK are expected when metformin, pravastatin, and rosuvastatin are administered concomitantly with dasatinib.

## Supplementary Information

Below is the link to the electronic supplementary material.

Supplementary file1 (PDF 762 KB)

## Data Availability

Bristol Myers Squibb policy on data sharing may be found at https://www.bms.com/researchers-and-partners/independent-research/data-sharing-request-process.html.
