# Supplementary material for: Prediction of drug–drug interaction potential mediated by transporters between dasatinib and metformin, pravastatin, and rosuvastatin using physiologically based pharmacokinetic modeling
Source: Cancer Chemother Pharmacol. 2022 Feb 11;89(3):383–92. doi: 10.1007/s00280-021-04394-z (PMC8882081; doi:10.1007/s00280-021-04394-z)
Supplement: Supplementary file 1 — Supplementary file1 (PDF 762 KB) [file 280_2021_4394_MOESM1_ESM.pdf]

Supplementary Material

Prediction of drug–drug interaction potential mediated by transporters between dasatinib and metformin, pravastatin, and rosuvastatin using physiologically based pharmacokinetic modeling

Ming Chang,* Sai Bathena, Lisa J. Christopher, Hong Sheng, Amit Roy

Bristol Myers Squibb, Princeton, NJ 08540, USA

*Corresponding author

Ming.Chang@bms.com

Journal: *Cancer Chemotherapy and Pharmacology*

Supplementary Table 1 Summary of preclinical and clinical studies used to support the model development and evaluation

Study number	Title	Reference
	Preclinical studies	
NCPK583/ DCN930147085	This study was conducted to investigate the potential for dasatinib to inhibit the MATE2K transporter.<br><br>For this experiment, a stable HEK cell line over-expressing MATE2K was used. The effects of dasatinib on probe substrate ([^{14}C]metformin, 2 μM) uptake into the transporter-expressing cell line relative to the HEK parental (mock) cells was evaluated. The targeted final test concentrations of dasatinib were 50, 16.7, 5.56, 1.85, 0.617, 0.206, and 0.0686 μM. Incubations were conducted in triplicate. Pyrimethamine was used as positive control inhibitor and tested over the concentration range of 0.00686 to 5 μM.<br><br>Stably transfected MATE2-K/HEK and Mock/HEK cells were seeded in 24-well poly-D-lysine-coated Biocoat plates (BD Biosciences, Franklin Lakes, NJ) at a density of 500,000 cells/well. The cells were grown in supplemented DMEM cell culture medium and formed monolayers on the bottom of wells. The cells/plates were ready to use 2 days after seeding. [^{14}C]Metformin, dasatininb and pyrimethamine, were freshly prepared in Hanks' balanced salt solution (HBSS) buffer supplemented with 10 mM N-2-hydroxyethylpiperazine-N'-2-ethanesulfonic acid (HEPES, pH 8.4).<br><br>Prior to use in the experiment, cell monolayers were washed twice with pre-warmed HBSS. Cells were then pre-incubated with buffer containing dasatinib or pyrimethamine for 30 minutes. After pre-incubation, solution was removed and incubation solutions containing [^{14}C]metformin and dasatinib or pyrimethamine were added to cell monolayers to initiate the incubations. At the designated time after the incubation, uptake buffer was removed and cells were rinsed 3 times with 1 mL of ice-cold HBSS buffer. Plates were then dried and cells were lysed with 0.1% Triton-X 100. Aliquots of cell lysate were used for radioactivity counting (liquid scintillation counter) and protein concentration analysis (BCA protein assay kit (Pierce, Rockford, IL) with ultraviolet (UV) spectral detection and albumin as the protein standard. Cellular uptake was normalized based on the protein amount in each well.	Internal study report

	Uptake of probe substrate, [^{14}C]metformin, was determined in both transporter-expressing cells and mock cells in the presence of a dasatinib or a positive control inhibitor (pyrimethamine). The transporter-mediated uptake was calculated after subtracting the uptake by mock cells from uptake by the transporter-expressing cells. All data were expressed as mean ± standard deviation (SD).<br><br>The inhibitory effect of dasatinib and pyrimethamine on transporter activity was expressed as the amount of [^{14}C]metformin uptake in milligrams per picomole per minute (mg/pmol/min) in the presence of dasatinib or pyrimethamine. To calculate IC_{50} values, the inhibition values were plotted against inhibitor concentration, then the inhibition-concentration curves were fitted by means of nonlinear least-squares regression analysis to determine the IC_{50}.	
NCPK601/ DCN930147497	The potential for dasatinib to inhibit Pgp and BCRP transporters was investigated using Caco-2 cells assay. The probe substrates used for the investigation were digoxin (Pgp substrate) and cladribine (BCRP substrate). Cyclosporin A and KO143 were used as positive control inhibitors for Pgp and BCRP, respectively. Stock solutions of all inhibitors and substrates were prepared in DMSO and diluted into buffer accordingly.<br><br>Cells were seeded onto collagen-coated polycarbonate filter membranes in 24-well Transwell plates. The cells were grown in fortified DMEM media maintained at 37°C in a 95% relative humidity and 5% CO_2 atmosphere. Before the experiment, the plated cells were evaluated for tight junction formation (via TEER measurement with an EVOM resistance meter, World Precision Instruments, Sarasota, FL). Each well of the plate demonstrated a TEER value of 600 Ω•cm^2, indicating suitability of the cells for the experiment. Before assay execution, each cell monolayer was washed 3 times with assay buffer (MHBSS containing 10 mM HEPES, pH 7.4) to remove culture media.<br><br>In the Digoxin Efflux Inhibition Assay, the bidirectional permeability of digoxin was monitored in the Caco-2 cells in the presence and absence of 10 concentrations of dasatinib (n=6 replicates) or cyclosporin A (positive control) and the percent inhibition of digoxin efflux was determined. The concentration of the digoxin, added to the donor compartment, was 5 μM. The final test concentrations of inhibitors, dasatinib and cyclosporin A (added to both donor/receiver compartments), were 50, 25, 12.5, 6.25, 3.13, 1.56, 0.78, 0.39, 0.195, and 0.098 μM.	Internal study report

	In the Cladribine Efflux Inhibition Assay, the bidirectional permeability of cladribine was monitored in Caco-2 cells in the presence and in the absence of 10 concentrations of dasatinib or KO143 (positive control) and the percent inhibition of cladribine efflux was determined. The targeted concentration of cladribine, added to the donor compartment, was 5 µM. The concentrations of dasatinib were 50, 25, 12.5, 6.25, 3.13, 1.56, 0.78, 0.39, 0.195, and 0.098 µM and the concentrations of KO143 were 10, 5, 2.5, 1.25, 0.63, 0.31, 0.16, 0.08, 0.04, and 0.02 µM. The inhibitors were added to both donor and receiver compartments.<br><br>The concentrations of digoxin and cladribine were determined by solid-phase extraction followed by tandem mass spectrometry (SPE-MS/MS) on an Agilent RapidFire™ HT-300 System and an API 4500 mass spectrometer from AB Sciex using positive ion electrospray and selected reaction monitoring (SRM). Efflux ratios (defined as a ratio of permeability coefficients B→A to that of A→B were determined. Percent inhibition of digoxin or cladribine efflux was determined by comparing the difference between the Pc values for a probe incubated in the presence and in the absence of dasatinib or control (cyclosporin A or KO143, respectively).<br><br>The results for all assays were expressed as percent inhibition of transporter activity. To calculate IC_{50} values, the inhibition values were plotted against inhibitor concentration, then the inhibition-concentration curves were fitted using a 4-parameter logistic regression model.<br><br>Cyclosporin A and KO143 were able to completely inhibit digoxin or cladribine efflux, respectively, in a concentration-dependent manner with IC_{50} values that were within their acceptable historical range (1 to 5 µM for cyclosporin A and 0.1 to 0.5 µM for KO143).	
NCPK601/ DCN930147497	This study was conducted to investigate the potential for dasatinib to inhibit OATP1B1, OATP1B3, MRP2, OAT1, OAT3, OCT2, MATE1 transporters.<br><br>Uptake transporters were evaluated using stable cell lines developed in an HEK293 cell background: each cell line heterologously over-expresses a single transporter of interest (OATP1B1, OATP1B3, NTCP, OAT1, OAT3, OCT1, OCT2, or MATE1). The effects of dasatinib on probe substrate uptake into each transporter-expressing cell line were evaluated relative to probe substrate uptake in HEK293 parental (mock) cells. The	Internal study report

probe substrates used in the evaluation were pitavastatin (0.3 μM, for OATP1B1), [^{3}H]CCK-8, 1.3 μM, OATP1B3), [^{3}H]estradiol-17ß-glucuronide, MRP2), [^{3}H]para-amino-hippurate 1.0 μM, OAT1), [^{3}H]estrone -3-sulfate 1.0 μM, OAT3), metformin 250 μM (OCT1), metformin 5 μM (OCT2), metformin 5 μM (MATE1). The targeted final test concentrations of dasatinib were 50, 16.6, 5.55, 1.85, 0.62, 0.206, 0.069, 0.023, 0.0076, and 0.0025 μM.

MRP2 inhibition was assessed using inside-out plasma membranes (vesicles) from baculovirus-infected insect cells expressing human MRP2. The effects of dasatinib on probe substrate uptake into inside-out vesicles were determined in the presence of ATP and evaluated relative to probe substrate uptake in the presence of AMP (background control).

Stock solutions of dasatinib and positive controls in 100% DMSO were diluted appropriately into a corresponding well of the cellular assay plate. After a 10-minute preincubation with dasatinib or control inhibitor, the appropriate substrates were added to each well of the assay plate. Assay plates were incubated at room temperature (20°C to 25°C) for 20, 40 or 60 minutes.

At the end of the incubation time, assay buffer was aspirated and cells were washed 3 times with modified HBSS.

In OATP1B1 assay, after final wash, cells were lysed with acetonitrile/water mixture (1:1, v/v) containing internal standard. The supernatant was then analyzed by SPE-MS/MS.

In OCT1/2 and MATE1 assays, after final wash, cells were lysed by adding acetonitrile containing [$^{2}H_{6}$]metformin as internal standard. The supernatant was analyzed by LC-MS/MS.

In OATP1B3 and OAT1/3 assays, after final wash, cells were lysed by adding lysis buffer (1% sodium dodecyl sulfate [SDS], 0.1 N sodium hydroxide in water). Following 60-minute incubation in cell lysis buffer, scintillation fluid was added to each well and radioactivity was counted using a microplate scintillation counter.

In MRP2 assay, partially-inverted plasma membrane vesicles from SF9 cells expressing MRP2 were diluted to a concentration of 2 mg/mL in assay buffer (50 mM MOPS-Tris, pH 7.4, 70 mM potassium chloride, 7.5 mM magnesium chloride) and added to the assay plate. Subsequently, ATP substrate solution (6.15 mM ATP, 7.7 μM glutathione, 100 μM [^{3}H]β-Estradiol-3-(β-D-glucuronide)-17-sulfate [6.25 μCi/mL] in assay buffer) or AMP

	substrate solution (6.15 mM AMP, 7.7 µM glutathione, 100 µM [3H]β-Estradiol-3-(β-D-glucuronide)-17-sulfate [6.25 µCi/mL] in assay buffer) was added to wells. The latter to define a minimal signal equivalent to 100% inhibition.<br><br>Assay plates were incubated at room temperature (20°C to 25°C) for 40 minutes and reactions were stopped by addition of ice-cold wash buffer (50 mM MOPS-Tris, pH 7.4, 70 mM potassium chloride). Reactions were transferred to 384-well glass-fiber filter plates, and then washed 7 times with ice-cold wash buffer. Filter plates were dried for 1 hour, then scintillation fluid was added to each well and plates were incubated for 1 hour prior to quantifying radioactivity using a microplate scintillation counter.<br><br>For OATP1B1, BSEP, NTCP, OCT1/2, and MATE1 assays, the signal intensity of the substrate in MS/MS analysis was normalized to the signal of internal standard; thus signal intensity was expressed as signal ratio. For radiometric assays the signal for each well was expressed as counts per minute. Each sample signal or signal ratio was then normalized to signal of the maximal control (uptake in the cell line expressing the transporter of interest in the presence of 0.5% DMSO, defined as 0% inhibition) and minimal control (uptake in the parental cell line HEK293 in the presence of 0.5% DMSO, defined as background or 100% inhibition).<br><br>The results for all assays were expressed as percent inhibition of transporter activity. Values were plotted and the IC_{50} values for each transporter were determined from fitted curves.	
DCN930011322	This study was conducted to investigate inhibition of CYP2C8 and CYP3A4 enzymes by dasatinib in pooled human liver microsomes (HLM). The probe substrates used for the investigation were paclitaxel (CYP2C8) and midazolam (CYP3A).<br><br>The ability of dasatinib to reversibly inhibit the activity of CYP2C8 was evaluated in pooled HLM (n=15, CellzDirect, NC). All incubations were conducted in duplicate. The marker substrate, paclitaxel, was used at concentrations of 1, 2, 5, 10, and 20 µM in the presence of 0.075 mg/mL microsomal protein in 100 mM potassium phosphate buffer (pH 7.4). Dasatinib was added to achieve final concentrations of 0, 3, 5, 10, 15, and 25 µM (bracketing the estimated IC_{50} value [12 µM]). Incubation mixtures were placed in a 37°C water bath for approximately three minutes prior to initiating the 10-minute incubation reaction with the	Internal study report

addition of NADPH (1 mM final concentration). After 10 minutes, the reactions were terminated and extracted. The concentration of the 6-hydroxy metabolite was determined by LC-MS/MS. A Lineweaver-Burke plot of the data was prepared to determine the inhibition model that best-fit the experimental data and a Ki value for inhibition of CYP2C8 by dasatinib was calculated.

A detailed time and concentration-dependent experiment was performed to determine the kinetics parameters (K_I and k_{inact}) for the time dependent inactivation of CYP3A4 by dasatinib. Time dependent inactivation of CYP3A4 by dasatinib in human liver microsomes was tested by following a decrease in CYP3A4-catalyzed midazolam hydroxylation over time. Duplicate samples containing approximately 0.5 mg/mL protein, were pre-incubated for various time intervals (0, 5, 10, 15, 20, and 25 min) at 37°C with several concentrations of dasatinib (0, 2, 5, 10, and 25 μM), in the presence of 10 mM NADPH. After the pre-incubation period, the primary incubations were diluted 10-fold to produce samples for secondary incubations. Secondary reactions were initiated by addition of 10 μM midazolam, the marker substrate. The secondary incubation reactions were stopped after 4 minutes and extracted. Concentrations of marker metabolite, 1-hydroxy midazolam, were determined using a validated LC-MS/MS method. In order to demonstrate that the time dependent inhibition for dasatinib was NADPH dependent, control incubations containing 25 μM dasatinib were prepared which did not contain NADPH in the primary reactions.

The percent remaining activity after incubation for various times in the pre-incubation step at several concentrations of dasatinib were then plotted as the natural log of the mean % of control activity vs. pre-incubation time. The initial rate constant for enzyme inactivation (k_{obs}) at each concentration of dasatinib was estimated from the plot, where the slope of a linear regression line is $-k_{obs}$. The k_{inact} (the maximum rate constant for inactivation) and K_I (the inactivator concentration at half the maximal rate of enzyme inactivation) values were determined from linear regression of a double-reciprocal plot of the K_{obs} values versus the dasatinib concentrations where the y-intercept provided the value of $1/k_{inact}$ and the slope/intercept was equivalent to K_I.

Two known-mechanism based inhibitors, diltiazem, and erythromycin, were similarly analyzed and served both as positive

	controls and to provide a context for the time-dependent inhibition of CYP3A4 by dasatinib.	
DCN950068845	GastroPlus PBPK model was applied to two formulations and verified with clinically observed results.	Internal study report
	Clinical studies	
CA180249	This study was conducted to evaluate the effect of a 40-mg daily dose of omeprazole on the pharmacokinetics of a single oral 100-mg dose of dasatinib in healthy volunteers. See NCT00655746 for Study details, Tabular view, and Study Results.	https://clinicaltrials.gov/ ct2/show/results/ NCT00655746
CA180021	The phase 1 study was conducted to evaluate the effect of ketoconazole on the pharmacokinetics of dasatinib and the effect of dasatinib on pharmacodynamic markers in patients with advanced solid tumors. See NCT00162214 for Study details and Tabular view.	https://clinicaltrials.gov/ ct2/show/results/ NCT00162214
CA180002	A phase I dose-escalation study was conducted to determine the safety, pharmacokinetics, and pharmacodynamics of BMS-354825 in the treatment of patients with chronic, accelerated, or blast phase chronic myelogenous leukemia, or Philadelphia chromosome positive acute lymphoblastic leukemia who have hematologic resistance to imatinib mesylate (Gleevec®). See NCT00064233 for Study details and Tabular view.	https://clinicaltrials.gov/ ct2/show/results/ NCT00064233
CA180016	This was an open-label, randomized, four-arm, single dose, formulation comparability study of BMS-354825 in healthy adult volunteers to estimate the comparability of the 5 mg clinical form (Treatment B), and the 20 mg (Treatment C), and 50 mg (Treatment D) commercial dosage forms of BMS-354825 to the 50 mg clinical form (Treatment A). Eight-eight volunteers were enrolled to ensure at least 80 evaluable volunteers. Eligible volunteers reported to the study facility on the evening of Day-2. On Day -1, serial ECGs were performed at times approximating the schedule for Day 1. On Day 1, following an overnight fast of at least 10 hours, 88 volunteers were randomized to receive a single 100 mg dose of open label BMS-354825 (n=22 for each of the 4 study dosing regimens). Serial ECGs, physical examinations and vital signs were performed, and blood and urine for pharmacokinetics and for safety assessments were collected at selected times pre- and post-dose. The volunteers were confined to the study facility for the entire duration of the study. They were closely monitored for adverse events throughout the study period.	www.accessdata.fda.gov/drugsatfda _docs/nda/2006/ 021986s000_Sprycel __ClinPharmR.pdf

	They were discharged from the facility on Day 2, after all discharge procedures are completed.	
CA180009	This was an open-label, randomized, three-period, and three-treatment, crossover study evaluating the effect of a light-fat meal and a high-fat meal on the pharmacokinetics of BMS-354825 in healthy volunteers. Volunteers underwent screening evaluations to determine eligibility within 21 days prior to study enrollment. Volunteers were admitted to the clinical facility prior to dosing on Day -2 of Period 1. On Day 1 of Period 1, 54 volunteers were randomized to one of six sequences (ABC, ACB, BAC, BCA, CAB or CBA) according to a computer-generated randomization schedule. The three treatments were a single oral dose of 100 mg BMS-354825 in a fasted condition (Treatment A), 30 minutes after start of a standard light-fat breakfast (Treatment B) and after a standard high-fat breakfast (Treatment C). All volunteers were fasted at least 10 hours prior to treatment and 4 hours post treatment. For Treatments B and C, food was consumed within 30 minutes. All tablets were ingested within 10 minutes. The treatments were administered on Days 1, 8, and 15. (i.e. Day 1 of each treatment period). There were at least a 7-day washout period between each dose. Blood samples were collected for pharmacokinetic analysis up to 24 hours post-dose. Physical examinations, physical measurements with vital signs, 12-lead ECG, and clinical laboratory evaluations were performed at selected times throughout the study. Volunteers were closely monitored for adverse events throughout the study and were released from the treatment facility on Day 2 of each treatment period.	www.accessdata.fda.gov/drugsatfda _docs/nda/2006/021986s000_Sprycel __ClinPharmR.pdf
CA180022	This was an open-label, randomized, two-period, two-treatment, crossover study in healthy volunteers to assess the effect of 100 mg of BMS-354825 on the single dose pharmacokinetics of 80 mg simvastatin. Volunteers underwent screening evaluations to determine eligibility within 21 days prior to study enrollment. Volunteers were admitted to the clinical facility on Day -2 of Period 1. On Day 1 of Period 1, volunteers were randomized to one of two treatment sequences to receive each of the following two treatments:<br>Treatment A: Simvastatin 80 mg (single dose)	www.accessdata.fda.gov/drugsatfda _docs/nda/2006/021986s000_Sprycel __ClinPharmR.pdf

	Treatment B: Simvastatin 80 mg + BMS-354825 100 mg (both single doses)<br><br>Alternate treatments were administered in subsequent periods. Treatment periods were separated by a minimum 7-day washout period. Blood samples were collected for pharmacokinetic analysis for 24 hours post-dose for BMS-354825 and simvastatin. Physical examinations, vital sign measurements, 12-lead ECG, and clinical laboratory evaluations were performed at selected times. Volunteers were closely monitored for adverse events throughout the study.	
CA180032	This was a single-sequence design evaluating the effects of 8 days of oral dosing with 600 mg rifampin each evening on the pharmacokinetics of a single oral dose of BMS-354825 on the mornings of Days 1 and 9 in healthy volunteers. Volunteers underwent screening evaluations to determine eligibility within 21 days prior to study enrollment.<br><br>Volunteers were admitted to the clinical facility the evening of Day -2. Twenty volunteers were enrolled to ensure at least 15 evaluable volunteers. A single oral dose of 100 mg BMS-354825 ware administered to all 20 volunteers in the morning (9:00 am) on Days 1 and 9 (fasted). Rifampin was administered in the evening (9:00 pm) from Day 2 to Day 9. Volunteers were confined to the clinical facility until 24 hours post-dose. Blood samples were collected on Day 1 and Day 9 for pharmacokinetic analysis of BMS-354825 up to 24 hours post-dose. Physical examinations, vital sign measurements, 12-lead ECG, and clinical laboratory evaluations were performed at selected times throughout the study. Volunteers were closely monitored for adverse events throughout the study and discharged from the treatment facility on Day 10.	www.accessdata.fda.gov/drugsatfda<br><br>_docs/nda/2006/021986s000_Sprycel<br><br>__ClinPharmR.pdf

ATP, adenosine triphosphate, *AMP,* adenosine monophosphate, *BCRP* breast cancer resistance protein, *BSEP* bile salt export pump, *CDER* Center for Drug Evaluation and Research, *CYP*, cytochrome P450, *DMEM,* Dulbecco's modified eagle medium, *HEK* human embryonic kidney, *ECG* electrocardiogram, *LC-MS*, liquid chromatrography mass spectrometry, *MATE2K* multidrug and toxin extrusion protein, *MRP* multidrug resistance-associated protein, *NADPH,* nicotinamide adenine dinucleotide phosphate, *NTCP* sodium taurocholate co-transporting polypeptide, OAT *organic anion transporter, OATP* organic anion transporting polypeptide, *OCT* organic cation transporter, *Pgp* P glycoprotein 1, *SRM,* selected reaction monitoring, *TEER* transepithelial electrical resistance

Supplementary Table 2 Studies used for base-model evaluation

Study	Purpose/Question	Treatment/Intervention(s)	Population (*N*)
CA180009	Food effect	Dasatinib 100 mg + fasting/high- or low-fat meal	HPs (49)
CA180016	Formulation comparability	Dasatinib 100 mg (2×50-mg/5×20-mg tablet)	HPs (19)
CA180032	Rifampin DDI	Dasatinib 100 mg	HPs (20)
CA180249	Omeprazole DDI	Dasatinib 100 mg	HPs (14)
CA180002	Multiple ascending dose	Dasatinib 75 mg QD with 5 days on/2 days off	Patients (3)

DDI drug–drug interaction, *HP* healthy participant with evaluable pharmacokinetics, *QD* once daily

Supplementary Table 3 Simulation design and conditions

Simulation	Design/Conditions	Comment
**Single-dose PK**		
Dosing	Dasatinib tablet (100-mg single dose)	
Virtual trial	10 trials, 20 HPs/trial	Sample size and demographics referred to the four clinical trials used for comparison
Demographics	18–50 years and 0% female	
**Multiple-dose PK**		
Dosing	Dasatinib tablet (75 mg QD)	5 days on/2 days off for 4 weeks
Virtual trial	10 trials, 3 HPs/trial	Sample size and demographics referred to CA180002 used for comparison
Demographics	18–50 years and 0% female	
**Ketoconazole DDI**		
Dosing	Dasatinib 20-mg single dose on day 8/ketoconazole 200 mg BID on days 2–9	Referred to CA180021 (Supplementary Table 1)
Virtual trial	10 trials, 20 patients/trial	
Demographics	20–65 years and 50% female	
**Rifampin DDI**		
Dosing	Dasatinib 100-mg single dose on day 9/rifampin 600 mg QD on days 2–10	Referred to CA180032 (Supplementary Table 1)
Virtual trial	10 trials, 20 HPs/trial	
Demographics	18–50 years and 0% female	
**Simvastatin DDI**		
Dosing	Simvastatin 80-mg single dose on day 8/dasatinib 100 mg QD on days 3–9	Referred to CA180022 (Supplementary Table 1)
Virtual trial	10 trials, 20 HPs/trial	
Demographics	18–50 years and 0% female	
**Metformin DDI**		
Dosing	Metformin 500-mg single dose on day 1 at 9 a.m./dasatinib 100-mg single dose on day 1 at 8 a.m.	
Virtual trial	10 trials, 10 HPs/trial	
Demographics	18–50 years and 0% female	
**Pravastatin DDI**		
Dosing	Pravastatin 40-mg single dose on day 8/dasatinib 100 mg QD on days 2–9	
Virtual trial	10 trials, 10 HPs/trial	
Demographics	18–50 years and 0% female	

**Rosuvastatin DDI**	
Dosing	Rosuvastatin 20-mg single dose on day 8/dasatinib 100 mg QD on days 2–9
Virtual trial	10 trials, 10 HPs/trial
Demographics	18–50 years and 0% female

BID twice daily, *BMS* Bristol Myers Squibb, *DDI* drug–drug interaction, *HP* healthy participant, *PK* pharmacokinetics, *QD* once daily

Supplementary Table 4 Comparison of simulated versus observed PK parameters following a single oral dose of 100 mg dasatinib in healthy participants

	**CA180009 (*N* = 49)**	**CA180016 (*N* = 19)**	**CA180032 (*N* = 20)**	**CA180249 (*N* = 14)**	**Simulated**
C_{max} (ng/mL) GM (CV%)	92 (50)	63 (63)	86 (36)	66 (51)	80 (40)
AUC (ng·h /mL) GM (CV%)	304 (47)	280 (49)	294 (33)	249 (46)	307 (42)
$T_{1/2}$ (h) Mean (SD)	4.84 (2.16)	4.45 (1.98)	4.74 (1.58)	4.00 (1.35)	4.73 (1.72)
T_{max} (h) Median (min, max)	1.0 (0.5, 3.0)	1.0 (0.3, 4.0)	1.0 (0.5, 3.0)	0.75 (0.5, 1.92)	0.79 (0.38, 1.73)
C_{max} ratio of simulated/observed	0.87	1.27	0.93	1.23	
AUC ratio of simulated/ observed	1.01	1.10	1.04	1.23	

AUC area under the concentration–time curve, C_{max} maximum observed concentration, *CV* coefficient of variation, *GM* geometric mean, *max* maximum, *min* minimum, *PK* pharmacokinetic, *SD* standard deviation, $T_{1/2}$ half-life, T_{max} time to reach maximum observed concentration

Supplementary Table 5 Dasatinib PBPK model validation using clinical DDI trials: ketoconazole, rifampin, and simvastatin

	**Dasatinib**	
	GMR of C_{max} (90% CI)	GMR of AUC (90% CI)
**Co-administered with ketoconazole**		
Observed	3.56 (2.84–4.44)	4.84 (3.83–6.13)
Simulated	2.70 (2.64–2.79)	4.93 (4.75–5.12)
Simulated/observed	0.76	1.02
**Co-administered with rifampin**		
Observed	0.19 (0.16–0.29)	0.16 (0.13–0.19)
Simulated	0.17 (0.16–0.18)	0.14 (0.13–0.15)
Simulated/observed	0.89	0.78
**Co-administered with simvastatin**		
Observed	1.36 (1.19–1.57)	1.23 (1.10–1.37)
Simulated	1.27 (1.26–1.29)	1.32 (1.30–1.34)
Simulated/observed	0.93	1.07

AUC area under the time–concentration curve, *CI* confidence interval, C_{max} maximum observed concentration, *DDI* drug–drug interaction, *GMR* geometric mean ratio, *PBPK* physiologically based pharmacokinetic

Supplementary Fig. 1 Comparison of simulated and observed plasma concentration–time profiles of dasatinib following a single oral dose of 100 mg. a. Linear scale, b. Log-linear scale

A

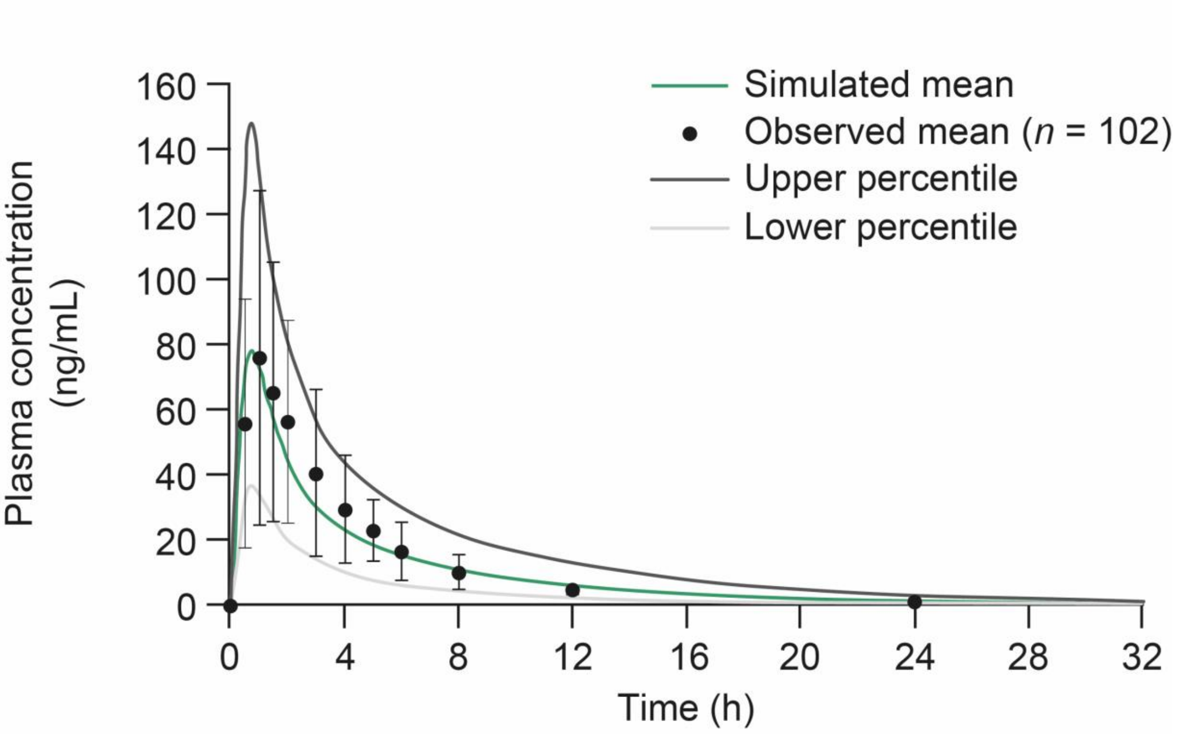

B

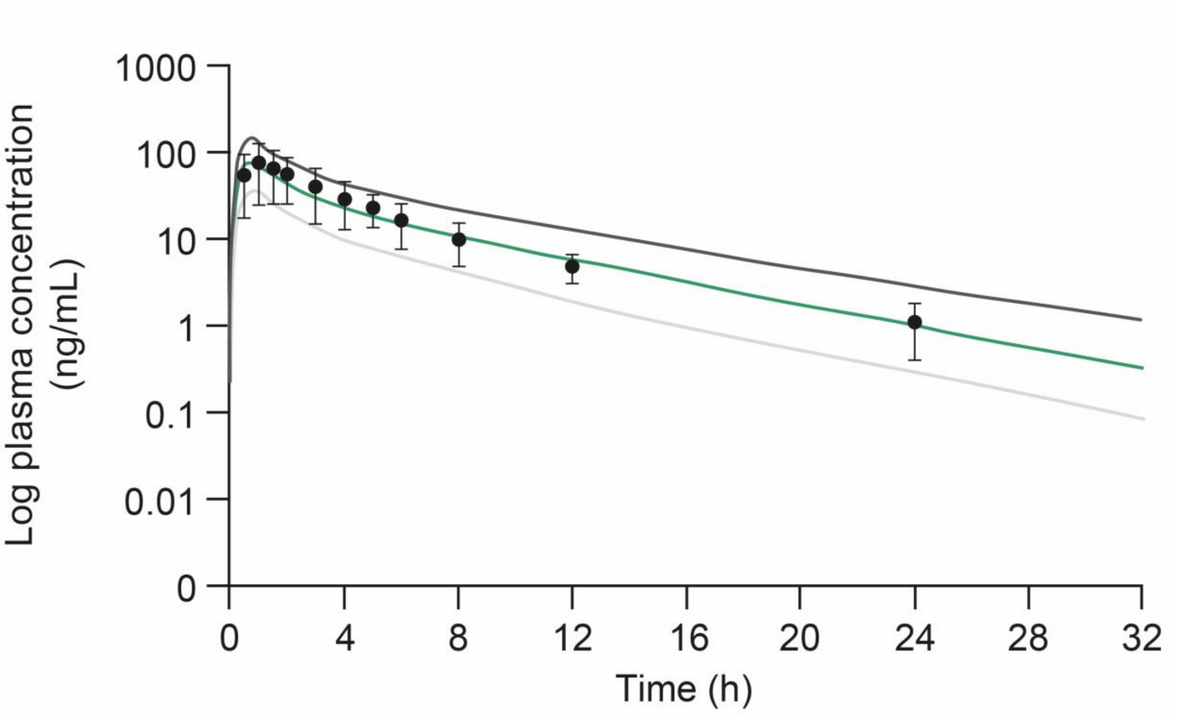

Observed mean plasma concentrations were the mean value of 102 healthy participants from four independent trials (CA180009, CA180016, CA180032, CA180249 [Supplementary Tables 1 and 2]) who received a single oral dose of 100-mg dasatinib under fasted conditions. Error bars represent standard deviations.

Supplementary Fig. 2 Comparison of simulated and observed plasma concentration–time profiles of dasatinib following 75 mg QD with a 5-day on/2-day off schedule. a. Linear scale, b. Log-linear scale

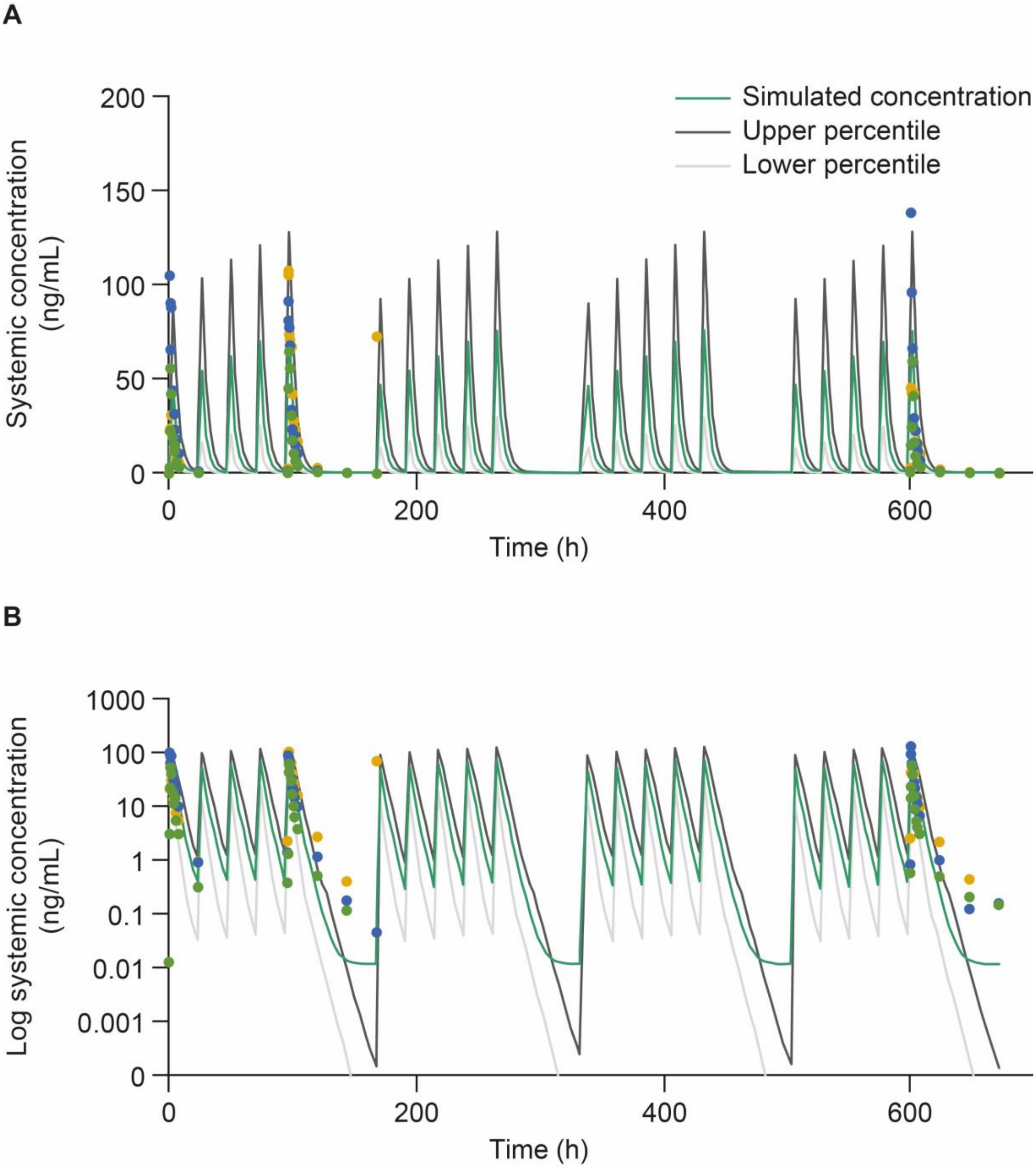

QD once daily

The three patients are represented by solid circles, color coded by individual. Observed plasma concentrations are from CA180002 (Supplementary Tables 1 and 2)

Supplementary Fig. 3 Comparison of simulated and observed mean plasma concentrations of a. dasatinib versus time with and without co-administration of ketoconazole, b. dasatinib versus time with and without co-administration of rifampin, and c. simvastatin versus time with and without co-administration of dasatinib

A

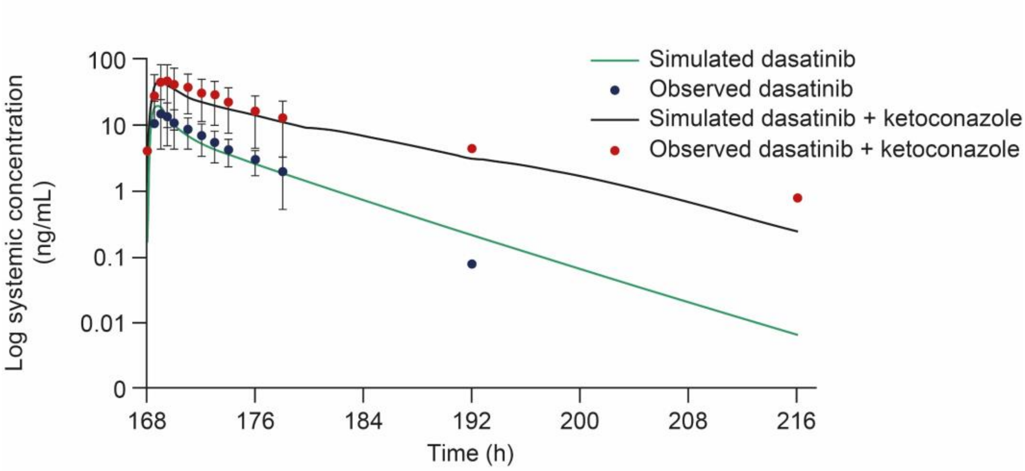

B

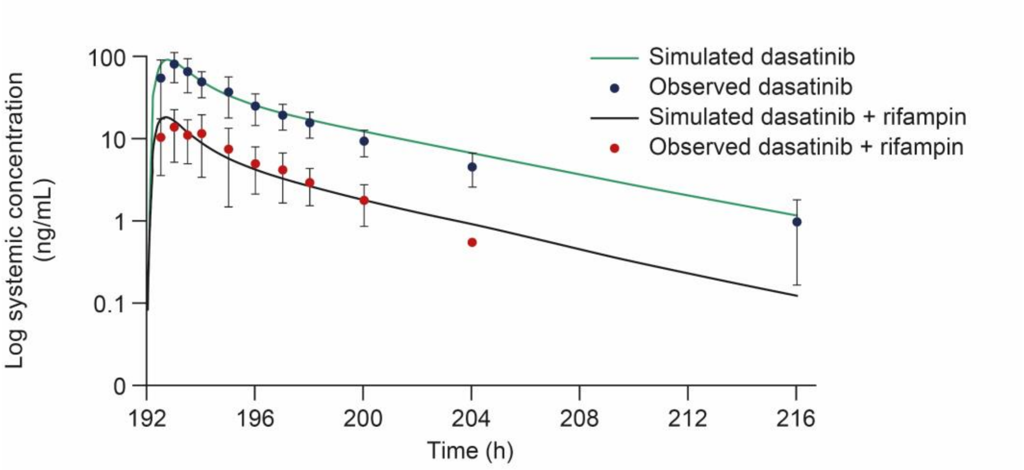

C

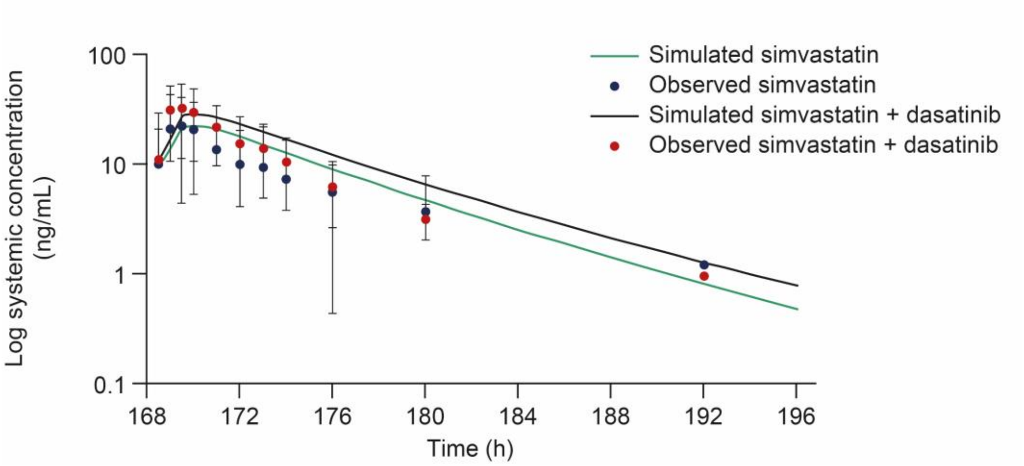

Observed mean plasma concentrations were taken from a. CA180021, b. CA180032, and c. CA180022 (Supplementary Tables 1 and 2). Error bars represent standard deviations.

Supplementary Fig. 4 Predicted mean plasma concentration–time profiles of a. pravastatin and b. rosuvastatin

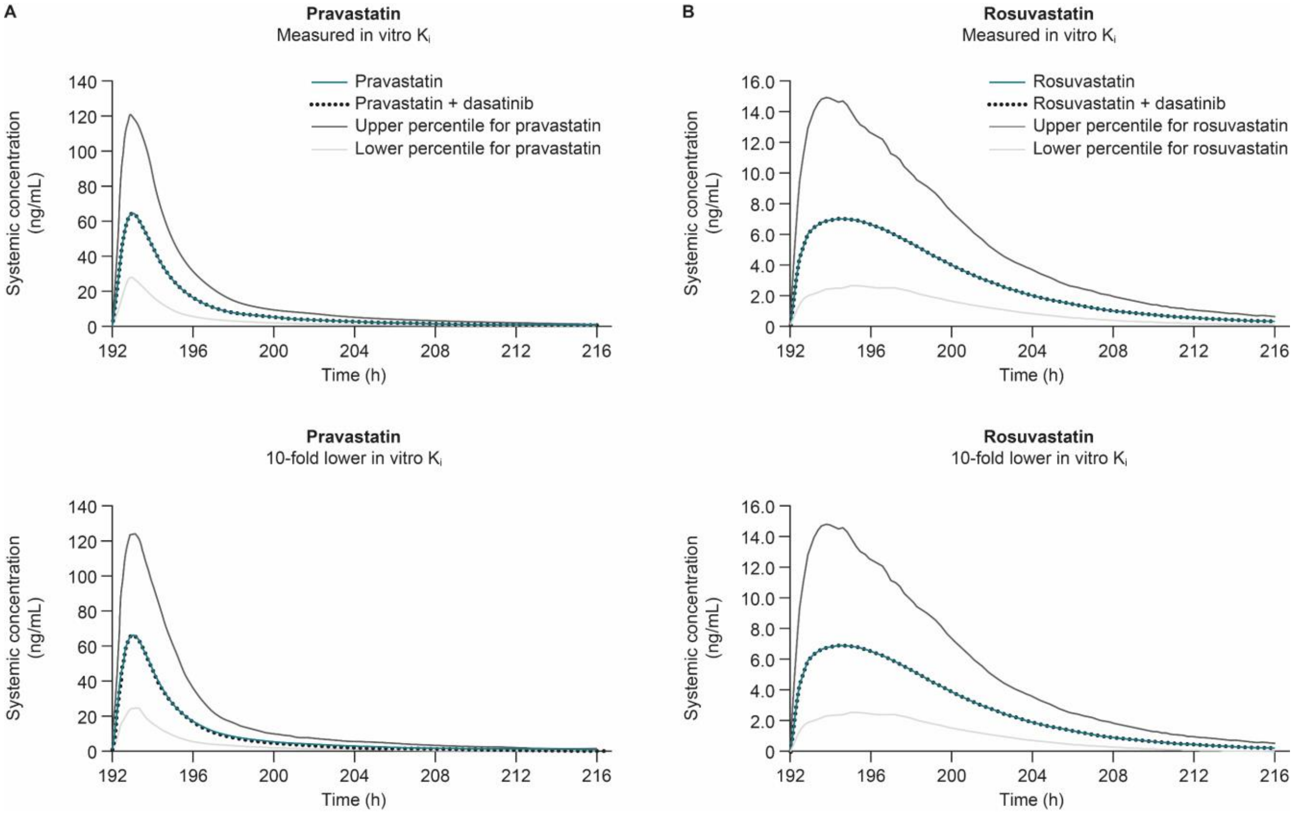

K_i inhibitor constant
